# Detection of the Progression of Anthesis in Field-Grown Maize Tassels: A Case Study

**DOI:** 10.34133/2021/4238701

**Published:** 2021-03-03

**Authors:** Seyed Vahid Mirnezami, Srikant Srinivasan, Yan Zhou, Patrick S. Schnable, Baskar Ganapathysubramanian

**Affiliations:** ^1^Department of Mechanical Engineering, Iowa State University, Ames, Iowa 50011, USA; ^2^Department of Agronomy, Iowa State University, Ames, Iowa 50011, USA; ^3^School of Computing and Electrical Engineering, Indian Institute of Technology Mandi, Kamand, H.P., India

## Abstract

The tassel of the maize plant is responsible for the production and dispersal of pollen for subsequent capture by the silk (stigma) and fertilization of the ovules. Both the amount and timing of pollen shed are physiological traits that impact the production of a hybrid seed. This study describes an automated end-to-end pipeline that combines deep learning and image processing approaches to extract tassel flowering patterns from time-lapse camera images of plants grown under field conditions. Inbred lines from the SAM and NAM diversity panels were grown at the Curtiss farm at Iowa State University, Ames, IA, during the summer of 2016. Using a set of around 500 pole-mounted cameras installed in the field, images of plants were captured every 10 minutes of daylight hours over a three-week period. Extracting data from imaging performed under field conditions is challenging due to variabilities in weather, illumination, and the morphological diversity of tassels. To address these issues, deep learning algorithms were used for tassel detection, classification, and segmentation. Image processing approaches were then used to crop the main spike of the tassel to track reproductive development. The results demonstrated that deep learning with well-labeled data is a powerful tool for detecting, classifying, and segmenting tassels. Our sequential workflow exhibited the following metrics: mAP for tassel detection was 0.91, F1 score obtained for tassel classification was 0.93, and accuracy of semantic segmentation in creating a binary image from the RGB tassel images was 0.95. This workflow was used to determine spatiotemporal variations in the thickness of the main spike—which serves as a proxy for anthesis progression.

## 1. Introduction

Flowering time in plants is geographically adapted. After a plant has been introduced into a new environment, flowering time can vary greatly [1, 2]. For field crops such as maize, date of flowering is crucial for yield. Therefore, altering reproduction time to achieve better adaptation to local environments and different climate conditions has become a major task in plant breeding. For example, breeders in the corn-planting regions of the North Central United States have found late maturity to be associated with higher yield, although overdelayed maturity might lead to yield loss caused by frost in early autumn [3, 4]. Maximum yields are typically achieved when the latest maturing hybrids that still mature prior to frost are deployed.

Successful seed production requires that male and female flowering be synchronized and that sufficient pollen is produced to fertilize all available ovules. Within a given farmer's production field, this is typically not a problem. In contrast, when a seed company seeks to produce a hybrid seed by crossing two inbred lines, one “male” and one “female” planning is required to ensure appropriate “nicking” of the two parents. But in addition to nicking, the duration of pollen production and the amount produced per day by the male genotype are important. In maize, anthesis typically begins ~2/3 of the way down from the maize spike of the tassel and then progresses upwards and downwards over the course of several days [5]. Understanding the genetic basis of the pattern of anthesis may offer a means to improve pollination efficiency in hybrid production. To do so, it is necessary to monitor the progression of anthesis under field conditions across diversity panels. Studies seeking to understand maize flowering patterns of limited numbers of genotypes have been performed using pollen traps [6, 7], which resulted in the development of a pollen dispersion model and for predicting total pollen shed. However, monitoring pollen shed in this manner on hundreds or thousands of genotypes under field conditions would be challenging.

The availability of lightweight, robust, and cheap imaging devices and associated developments in image processing has created the possibility of phenotyping faster and more accurately. Wheat flowering stages and ear emergence were monitored using computer vision in [8]. Similar methods have been implemented in automatically detecting flowering using time series RGB images taken in rice fields [9]. Image processing algorithms for extracting information from digital RGB images on color image segmentation in an HSI color space were developed and used to investigate Lesquerella flowering [9]. In these studies, flowering was divided into three stages: fully, partially, and nonflowering, and advanced image processing approaches were used [10]. [11] detected corn tassels under outdoor field conditions using image processing methods. [12] used an image-based bag-of-features to detect and locate maize tassels and also used advanced image processing approaches to extract features of individual tassels. Some tassel traits, including tassel and main spike length as well as tassel weight, were estimated using a heuristic image processing method called TIPS [13]. [14, 15] measured sorghum panicle length and width using lightbox images. These examples all demonstrate that using cameras and image processing tools allows vastly improved plant phenotyping. While successful, these classical methods have limited utility and have thus been used to only measure coarse features, or deployed under controlled, indoor conditions. This is attributed to a lack of robustness, especially when used on image data from field experiments (in contrast to lab experiments with tightly controlled imaging conditions). This is because natural factors, including weather, temperature, humidity, and wind speed, significantly affect image quality in the field, in addition to occlusion. This large variability makes it difficult (if not impossible) for purely image processing-based approaches to perform consistently.

In this context, the advent of deep learning (DL) approaches has revolutionized feature extraction from image data, specifically image classification [16], object detection [17], and segmentation [18]. Additionally, DL methods exhibit enhanced robustness and processing speed compared to conventional image processing. DL approaches have been successfully applied to an increasing array of problems in the plant sciences including precision agriculture [19], root segmentation [20], fruit detection [21, 22], sorghum detection and counting [23], fruit counting [24], weed detection [25], crop damage detection [26], disease classification [27], and disease detection [28]. More importantly, there have been several plant science-specific developments of deep learning workflows. For instance, a network called TasselNet was able to accurately count the number of maize tassels in a field [29]. [30] demonstrated a phenotyping system for automatically identifying northern blight-infected maize plants based on field image data. [31] used deep learning for estimating soybean leaf coverage in the field. StalkNet was developed to measure the sorghum stalk count and its width using Faster R-CNN and semantic segmentation [32]. The Panicle-SEG model developed a robust segmentation algorithm for a sorghum panicle using CNN and clustering superpixels [33]. [34] used CNN-based architectures to develop a wheat disease diagnosis system in a field environment. These developments have allowed breeders and biologists to collect and efficiently analyze image data from large fields and consider more complex phenotypic traits [35].

The careful integration of large field experiments with DL approaches opens up the possibility of quantitatively extracting spatiotemporal patterns associated with maize anthesis. This is the primary motivation of the current work. This is a challenging problem, with very limited studies—at the field scale—on temporal-spatial extraction of traits. We specifically focus on designing workflows to extract the spatiotemporal dynamics of flowering starting position and periodical flowering patterns on tassels based on a large-scale field experiment involving 500 consumer cameras that image a specific group of plants every 10 minutes. We describe development of a high-throughput system for recognizing the flowering patterns of different maize tassel genotypes, including design, development, and implementation of an automated end-to-end pipeline for monitoring such patterns. In the pipeline, various computer vision algorithms and heuristic image processing approaches were implemented. We emphasize that this approach is in contrast to a truly end-to-end framework that maps images directly to segmented tassel masks (say, by using an approach like Mask R-CNN [36]). While conceptually attractive, this was extremely difficult to implement due to the large dataset needed to train such models. Specifically, creating a viable dataset of semantically segmented images out of RGB images was extremely hard for this experiment due to the tassel structure. We therefore choose to decompose the task into independent detection and segmentation tasks, where either training datasets were easier to create by human labelers or an ensemble of standard image processing tasks work reasonably well.

## 2. Materials and Methods

### 2.1. Plant Materials

**Figure 1:**
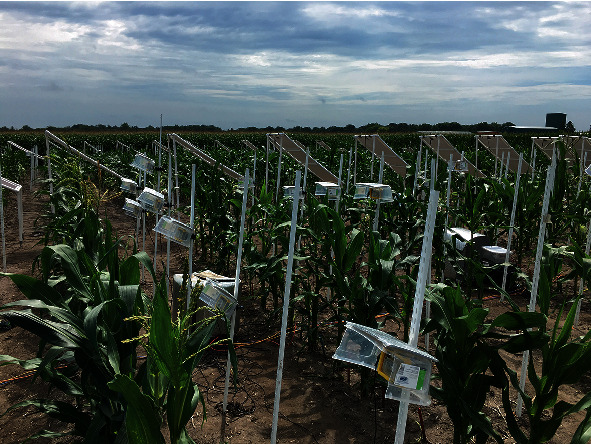
A photo of the experiment.

Leveraging availability of inexpensive 20 megapixel cameras integrated with advances in Internet of Things (IoT) that allows coordination of such cameras via cheap raspberry pi microprocessors, the Schnable group at Iowa State University deployed a large-scale plant image capture experiment during the summer of 2016 (Figure 1). Field-based phenotyping was conducted on inbred lines from two mapping populations, the Shoot Apical Meristem (SAM) diversity panel [37] and the Nested Association Mapping (NAM) population [38]. Genotypes in these two populations that exhibited compact tassel architectures were excluded from this study due to the difficulty in isolating tassel branches and central spike in images. Therefore, in total, 1117 genotypes, 185 from the SAM panel and 932 NAM lines, respectively, were phenotyped.

Genotypes were organized into four ranges with each genotype represented by a single plant. Each range contained 18 rows spaced 1.52 m (5 feet) apart. In each row, 16 plants were planted in an east-to-west direction at a 38 cm (15 inches) spacing, with border plants planted at the ends of each row. The field was organized first by population. Within each population, genotypes were ordered by plant height (using prior knowledge), and then, the planting order was randomized within each row of 16 plants. To minimize potential interference of plants behind any given row being imaged, the field was laid out such that shorter rows of plants were planted towards the north side of the field and thus a shorter row was always the background of any row being imaged. Using prior knowledge, genotypes were planted in descending order of height from south to north to minimize interference from background tassels in the captured images.

Images were collected using 456 cameras, with each camera imaging two plants, each of a different genotype. Each camera took an image every 10 minutes during a three-week growing period in August 2016. Due to the limited number of cameras available for field deployment, the cameras were used to collect images in early flowering genotypes from the SAM panel; once anthesis had been completed for the SAM panel, these cameras were redeployed to collect images of the NAM lines. Cameras were powered by solar panels.

### 2.2. Image Acquisition

For each pair of plants within a row, a pole-mounted Nikon Coolpix S3700 camera was positioned 60 cm south of the row. The camera was mounted facing the midpoint between two plants in the row, and the height of the camera was adjusted to match the height of the tip of the flag leaf of the shortest of the two plants to enable the camera to image both tassels. For cases in which two tassels had emerged at different times or at very different heights, the camera was adjusted so it could at least monitor anthesis of the earlier emerging tassel. The camera used for imaging each set of plants was placed south of the row with the camera facing north, to prevent overexposure due to the direct incidence of the sun on the camera. This camera orientation resulted in images consisting of pairs of tassels with the sky as the background.

**Figure 2:**
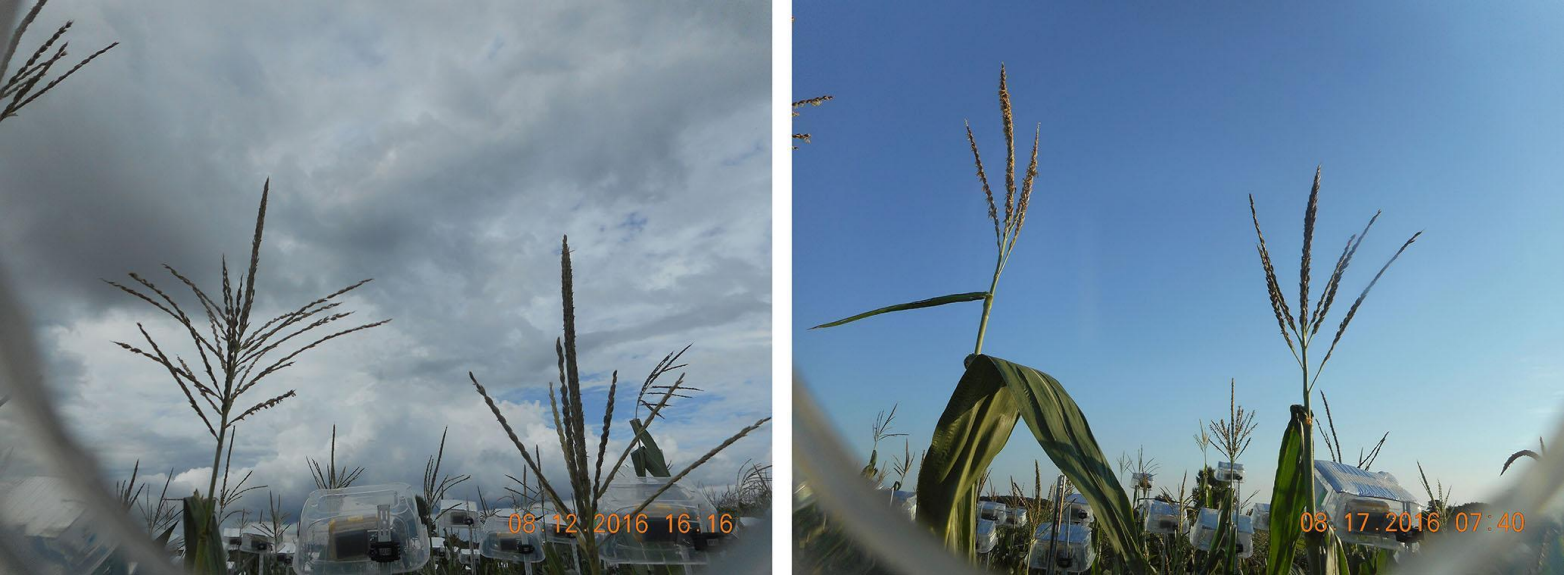
Two sample images of two different cameras.

**Figure 3:**
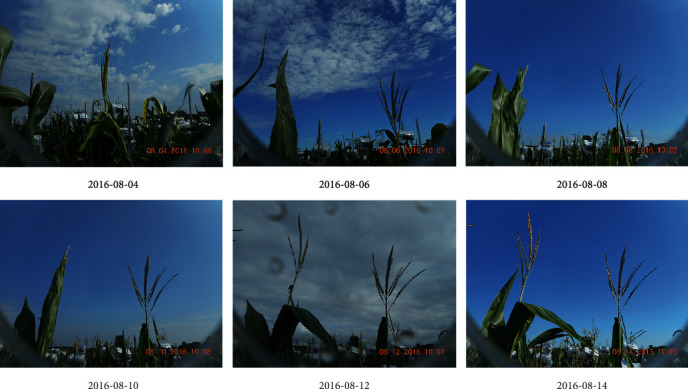
The first images taken after 10 am on the mentioned dates for a specific camera.

Every set of four cameras was connected to one Raspberry Pi2 processing unit, with four processing units powered by one solar panel and controlled by custom-written Python codes (unpublished) to produce images at 10-minute intervals from 7 am to 7 pm, starting after the first tassel had emerged from the whorl and terminating when anthesis of both plants monitored by a given camera was completed. Because tassels developed every day, the images were checked daily to ensure the tassels were not out of frame.  Figure 2 shows sample images from two different cameras, and the development progression of tassels as recorded by one camera is shown in Figure 3.

### 2.3. Workflow Overview

**Figure 4:**
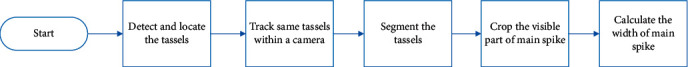
The steps of the entire end-to-end process.

We next describe the end-to-end pipeline used to analyze the tassels and monitor the flowering patterns. We divide the workflow into several steps, as illustrated in Figure 4. The first step is to *detect a tassel* in each image and *track its location* across the time sequence of image data. After detecting the tassel, the next step is to *segment* the tassel from the background to create a binary mask (i.e., black-white image). Finally, this time series of binary images is analyzed using image processing approaches to *extract physiologically meaningful traits*. The complete data is split into training, testing, and validation sets once. All subsequent steps used data from these nonintersecting datasets for training/testing/validation. Each of these steps is described in detail in the following sections.

### 2.4. Tassel Detection

**Figure 5:**
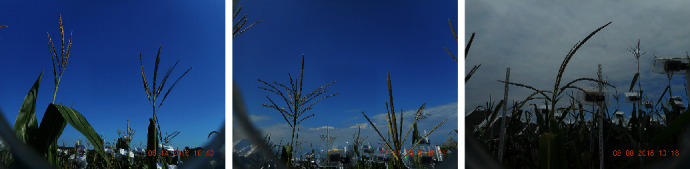
Different maize genotypes create various tassel structures.

Images captured over the growing season in Ames, Iowa, exhibited a wide variability and diversity. The first reason for such variability is weather—with foggy, rainy, sunny, and cloudy conditions impacting the image quality. Secondly, the imaging protocol was designed to contain two tassels in each camera imaging window. However, due to wind and ensuing occlusion, images contained two, one, or no tassels. Moreover, the presence of other objects in the imaging window, including leaves and other cameras, makes detection more difficult. Thirdly, each genotype exhibits a distinct (and diverse) tassel architecture. Finally, tassels developed during the imaging process, and the first image of a tassel was sometimes completely different from the last image of the same tassel.  Figure 5 illustrates genotypic variability using a small set of images captured from different cameras, making the case for a robust detection method to accurately detect and locate individual tassels.

We train and deploy a deep learning-based detection method called RetinaNet, which is a powerful object detection method described in [39]. The output of the model is a set of box-shaped coordinates surrounding the target object, a tassel in the current study. While this model is very similar to the Faster R-CNN [40] model, the loss function in this model has been modified and its implementation optimized for better tassel detection (see SI for details).

**Figure 6:**
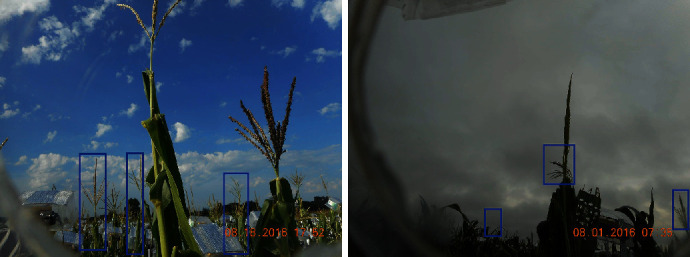
Examples of discarded annotations.

This method, like other supervised deep learning methods, required a training dataset. Images were first randomly selected from each camera, with a total of 3600 images selected from among more than 500,000 images. These images were annotated by human annotators using the Amazon Mechanical Turk tool. They were asked to draw a box on the two biggest tassels (i.e., the foreground) on the images, with an evaluation algorithm applied to the annotation to ensure accuracy and discard incorrect annotations (Figure 6; also, see supplementary information (available here)). Quality control of the annotated boxes included removal of very small boxes (measured in terms of pixel area), along with a check to see if the width and height of annotated boxes were within a priori defined bounds. Subsequently, images that passed these automated quality checks were visually checked. The final, quality assured, annotated dataset consisted of a total of 2911 images for analysis by RetinaNet, with 2500 of those used for training, 119 for validation, and 292 for testing. The model was trained using the dataset on the Nova cluster at Iowa State University using a GPU NVIDIA Tesla V100 with 32 GB memory. The details of the model as well as hyperparameter tuning are discussed in the supplementary information. The mean average precision (mAP) which is a standard metric for such problems is used to evaluate the accuracy of the model for object detection [41]. To do this, intersection over union (IOU) is calculated.

(1) $$\begin{array}{c} \text{IOU}=\frac{\text{area of overlap}}{\text{area of union}}. \end{array}$$

If IOU is more than 0.5, then the predicted box is marked true; otherwise, it is false. Then, the average precision of the model at different levels of recall was quantified to calculate the mAP.

### 2.5. Tassel Classification

**Figure 7:**
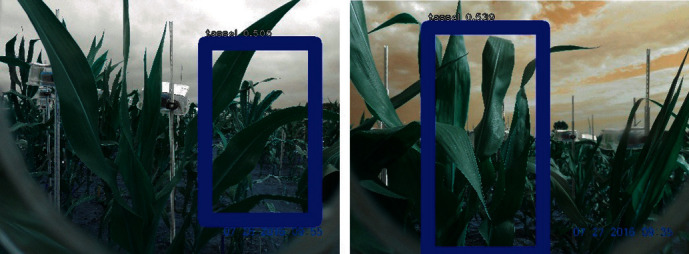
False-positive predicted boxes by the trained RetinaNet.

We perform additional quality assurance on the results produced by the tassel detection model. This is because, due to the wide diversity of images and imaging conditions, some false-positive boxes are predicted by the model (usually before tassel emergence because imaging may have started before emergence of tassels).  Figure 7 illustrates this by showing some of false-positive boxes predicted by the model.

A simple possibility to reduce false positives is to utilize the class confidence scores (output by the RetinaNet class subnet) and use a set threshold to classify detections as true tassels. However, extensive numerical exploration indicated that there was no good threshold—a high threshold produced a larger fraction of false negatives by missing tassels, while a lower threshold allowed false positives. To ensure some amount of redundancy, we keep the confidence threshold lower but apply another quality control network for checking (i.e., classification). Such smaller networks are significantly easier to train, can be reused, and have good performance. We train and deploy another (simple) model to differentiate between a box that contains a tassel and a box with no tassel. This tassel classification model then identifies the false-positive boxes predicted by the tassel detector and removes these data.

**Figure 8:**
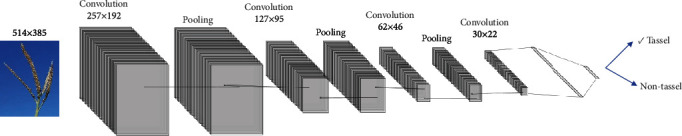
The classification model architecture.

We train and deploy a CNN-based binary classifier. Examples over the past few years suggest that CNN classification models produce robust classification models [16], especially for highly variable datasets characterized by a diversity of tassel shapes and weather as well as camera and leaf movements.  Figure 8 shows the architecture of the binary classification model using CNN. The input to the model is the image within the boxed region identified by the tassel detection step (Section 2.4), and the output of this model is a binary output (1 if the image is a tassel, 0 if it is not).

**Figure 9:**
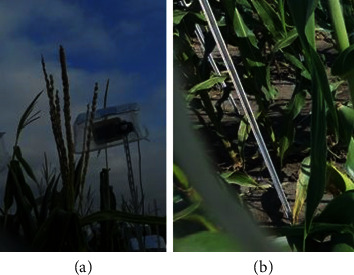
Sample images for training the classification model: (a) tassel class and (b) nontassel class.

The training dataset needed to train the model which was created by using 299 images from the boxes predicted by the object detection model. These images represent a diversity of tassel and leaf images, with 158 of the images belonging to the tassel class and 141 of the images belonging to the nontassel classes, with 270, 14, and 15 images selected for training, validation, and testing, respectively. Sample images for both classes are shown in Figure 9.

Since this is a binary classification, a confusion matrix based on the results of applying the trained model on the testing dataset was obtained and used to quantify the evaluation metrics, including precision, recall, accuracy, and F1 score. While “precision” quantifies how many of the detected boxes are actually tassels, “recall” quantifies how many of the tassels in the image have been identified. Model performance and robustness were decided based on the accuracy of the F1 score. These quantities are defined as follows:

(2) $$\begin{array}{c} \text{Precision}=\frac{\text{True Positive}}{\text{True Positive}+\text{False Positive}},\\ \text{Recall}=\frac{\text{True Positive}}{\text{True Positive}+\text{False Negative}},\\ \text{Accuracy}=\frac{\text{True Positive}}{\text{True Positive}+\text{False Positive}+\text{False Negative}},\\ \text{F}1\text{ score}=\frac{2\times \text{Precision}\times \text{Recall}}{\text{Precision}+\text{Recall }}. \end{array}$$

### 2.6. Tassel Tracking

Once tassels had been detected (and boxed), the next step is to track individual tassels within a given camera across the time series of images to enable extraction of temporal characteristics of anthesis. As mentioned earlier, the experiment was designed such that there were two tassels per image, one at the left and the other at the right side of the frame. But due to occasional movement of tassels due to wind, occlusion by leaves, or movement of camera position, the number of tassels and their relative locations were not consistent over time, requiring implementation of a tracking method to track and tag individual tassels in a camera across time. We utilized a simple strategy of associating the detected tassels in each image to a time series dataset corresponding to a genotype. For all the images from a specific camera that make up a time series of data, the centers of the two (biggest) detected boxes were considered (since there were two tassels imaged per camera and the tassels of interest were closed to the camera and hence had the largest size) followed by *K*-means clustering to cluster the points into two groups (*k* = 2). Boxes in the first cluster were associated with the fist tassel, and boxes in the second correspond to the second tassel. This approach—while seemingly simple—worked surprisingly well in tracking individual tassels across the time horizon.

### 2.7. Tassel Segmentation

**Figure 10:**
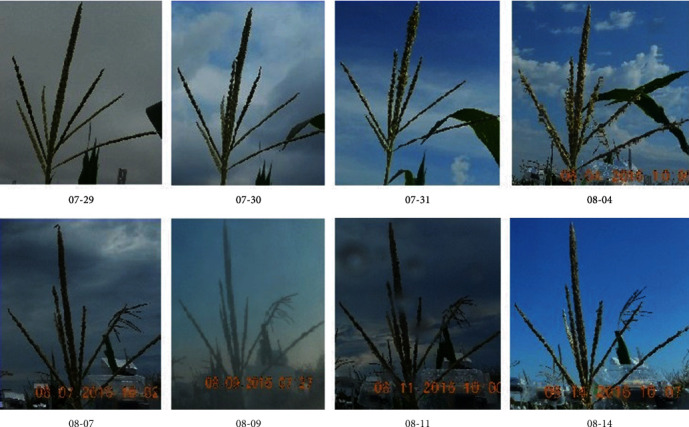
Tassel images with different backgrounds over time.

Following detection of the tassels as a function of time, the next step is segmenting out the tassel morphology from the image. We train and deploy a deep learning-based segmentation model, as the previous work [31] has shown the robustness of such approaches compared to regular thresholding approaches. Conventional thresholding strategies produced inconsistent results due to the diversity of backgrounds that includes sky, leaves, cameras, and tassels from the other rows. This is illustrated in Figure 10 that shows a sequence of time-lapse images tracking a single tassel, and it is clear that the background (as well as illumination) varies substantially. This representative image sequence shows effects of foggy, cloudy, sunny, or rainy weather.

**Figure 11:**
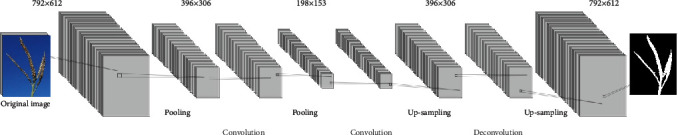
The segmentation model architecture.

In segmentation, an RGB image is mapped into a binary image pixel by pixel. We train and deploy a semantic segmentation method using a deep encoder-decoder model. Although meta-architectures such as Mask R-CNN [36] can be used to detect and segment the tassels simultaneously, at the time of writing this paper, we chose to go to the sequential approach using the RetinaNet. Our empirical experiments suggested that this approach performed better than Faster R-CNN, with the annotated dataset that we had. Additionally, our use of consensus-based image processing-based labeling with human quality control (see the next paragraph) provided an efficient approach to create an intermediate labeled dataset (we emphasize that architectures that are more recent could potentially outperform these results—and are the focus of the continuing work). This type of model consists of two symmetrical parts, the encoding section and the decoding section. The encoding section compresses the input image into a latent feature representation. The decoding section then uses this latent representation to map to a segmented image. The architecture of the deep encoder-decoder model is shown in Figure 11.

**Figure 12:**
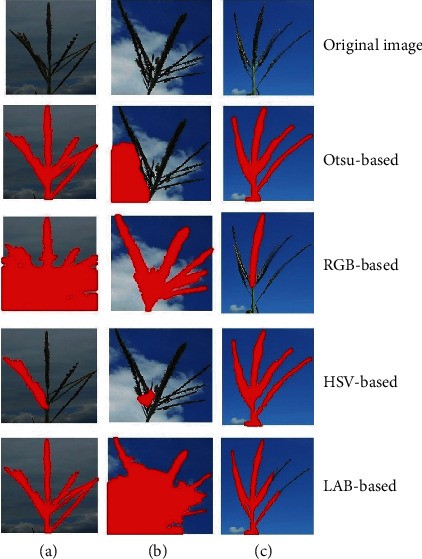
Three sample images with their corresponding segmentation. The red color shows the automatically annotated image with the corresponding segmentation method. An expert checked the images and selected the best segmentation mask or rejected all masks. For these images, Otsu, RGB, and HSV methods were selected for a, b, and c, respectively.

The model takes an RGB image as an input and produces the corresponding binary image (mask) as an output. We utilize a semiautomated approach to create the annotated dataset to train the model. Basically, we utilized four standard image segmentation approaches based on Otsu thresholding as well as thresholding based on conversion to RGB, HSV, and LAB color spaces [42]. While most image processing strategies worked reasonably, none worked well enough across a large fraction of the images to warrant usage. A human then selected the segmented images that most closely represented the true segmentation among the above-mentioned four image processing segmentation approaches. An expert performed a comparison of the four obtained masks for each image.  Figure 12 shows sample images with the four methods. If none of the masks were good, the image was discarded from the annotation dataset. This results in 1419 annotated images that were used for training (1000), validation (135), and testing (142). Such a strategy circumvents the need for human annotators to painstakingly segment out complex tassel shapes. Instead, we ask the human annotator to select the best segmented image created from a suite of image processing approaches. Such yes/no and multiple choice labeling of segmented data is cognitively much simpler than painstakingly segmenting image data [43]. We report the model accuracy and mean intersection over union (mIOU) that quantify the performance of the trained model works on the testing dataset [44]. mIOU is the average of intersection (common pixels in both the predicted and labeled) over union (all pixels in both the predicted and labeled).

### 2.8. Tassel Analysis

Once the segmented images are obtained, standard image processing methods were used to analyze the images. We are primarily interested in the flowering patterns (anthesis progression) of the central (or main) spike of the tassel. We define the main spike as the longest branch of the tassel. Since the main spike is sometimes occluded by other tassel features, we seek to extract the visible part of the main spike, which enables us to track anthesis progression. We divide the feature extraction into two steps: in the first step, we identify the longest branch in the tassel; in the second step, we identify the part of this longest branch which is the visible part of the main spike.

In the first step, we skeletonize the image [45], thus converting it into a graph that can be easily analyzed. The bottom tip of the graph is assumed to be the lower end of the tassel, after which all the possible end points (i.e., the end points of individual tassel branches) as well as paths were detected based on morphology methods [46]. The longest of these paths contains the main spike, and the corresponding end point to this path is the tassel tip.

**Figure 13:**
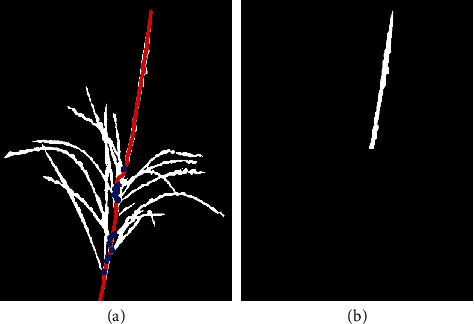
(a) The red line is the tassel path from the starting point to the tassel tip, and blue dots are the branch points. (b) The main spike of the tassel was cropped from the topmost branch point.

In the second step, we identify the branching points (i.e., locations along the longest path where secondary spikes start; see Figure 13). The main spike of the tassel is then the part of the path between the topmost branch point and the tip of the tassel. The tassel was finally cropped between the topmost branch point and the tassel tip. This object represents the visible part of the main spike. The width of this object at each pixel was obtained based on the method described in [14].

## 3. Results and Discussion

We show results and discuss the results of each step separately. This will enable nuanced assessment of each step. We finally illustrate the full pipeline for two representative camera data sequences.

### 3.1. Tassel Detection

**Figure 14:**
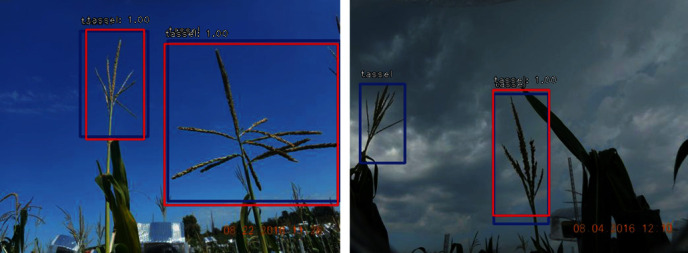
Predicted and ground truth boxes are shown in red and blue colors, respectively.

Transfer learning was used to train the RetinaNet object detection model to reduce the number of training epochs needed. The model was initialized with the ImageNet weights, and several training campaigns were deployed with different hyperparameters. The average time for training the model was approximately five hours, and the best model was chosen based on the higher mAP value obtained by testing the trained model on the unseen dataset, because the better-trained model explored features rather than memorizing a previously seen pattern. This model uses two loss functions, a regression and a classification loss function [39]. The first function is for bounding box regression, with value 0.078 after 100 epochs. The other is object classification to identify whether or not the object is a tassel, and its value was 0.0006 after 100 epochs. The mean average precision (mAP) values were used to check the robustness of the trained model on the testing dataset. RetinaNet returns the locations and probability of each detected box for each image, and mAPs were used for evaluating the model, producing a mAP value of 0.91.  Figure 14 shows sample predicted boxes in red and ground truth in blue, with mAP showing a notable match between the model and human annotation.

### 3.2. Tassel Classification

**Figure 15:**
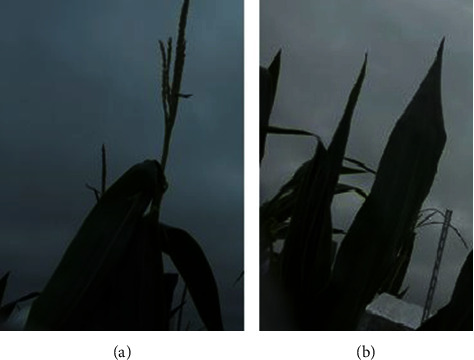
The classification model predicts (a) as a tassel and (b) as a nontassel.

After an exhaustive hyperparameter search of various convolutional neural network architectures, we chose the network shown in Figure 8. RGB images with sizes 387 × 516 × 3 pixels were used for inputs, and 28 3 × 3 filters were used for each of the convolutional layers. After each convolutional layer, there was a max pooling layer to reduce the computation load of the network and monitor important features. The activating function was the Rectified Linear Unit (ReLU). The 2D arrays were flattened to enable SoftMax to classify the images as a tassel or nontassel. A dropout [47] was also added to the model to prevent overfitting. The precision, recall, and F1 score of the model were 1, 0.875, and 0.93, respectively. Based on the results obtained, this model performed satisfactorily to check if the output of the RetinaNet model is valid.  Figure 15 shows two images predicted by RetinaNet and identified by the classification model as a tassel and nontassels.

### 3.3. Tassel Tracking

**Figure 16:**
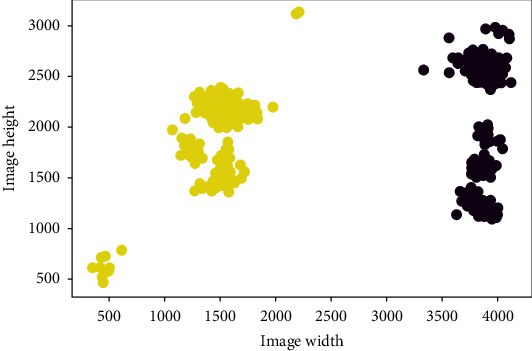
False-positive predicted boxes by RetinaNet.

Ideally, there would be two tassels per image captured by a camera. After tassel detection and classification, we must track the same tassel within a camera over the time period that the cameras are active. The cameras were checked daily in an effort to ensure that tassels were located within the frame.  Figure 16 is a scatter plot of the center points of tassels in a camera. These points were clustered using a *k*-mean clustering method with two clusters. The points in a cluster (yellow points or blue points in Figure 16) were grouped as the same tassel, and the images were cropped based on the box coordinates.

### 3.4. Tassel Segmentation

**Figure 17:**
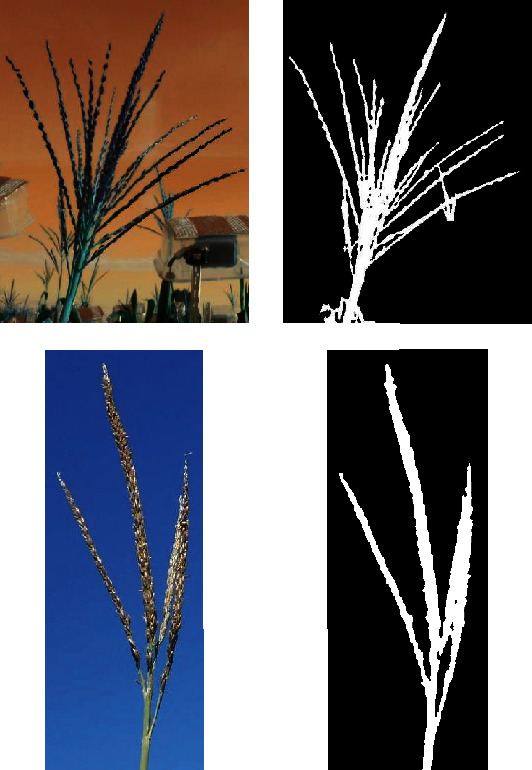
Two sample tassels predicted by RetinaNet (left side) and their corresponding binary images predicted by the segmentation model.

Extensive hyperparameter exploration was performed to identify a good CNN-based segmentation model. The final accuracy of the model was 0.95. In addition, the mIOU of the model is 0.69. The trained model was used to segment tassels from the background, producing as output a binary tassel image where the tassel is white and the background is black. A sample output image predicted by the model is shown in Figure 17.

### 3.5. Tassel Analysis

Image processing and morphological operations were performed after the binary images of tassels are obtained. The main spike was cropped based on the approach described in Materials and Methods. The lengths of the cropped main spike varied image by image because a branch's movement might occlude the main spike branch and make topmost branch point detection harder, so the width was calculated from the tip to the bottom of the cropped main spike to enable comparison with the width at the same branch location.

#### 3.5.1. Flowering Pattern

**Figure 18:**
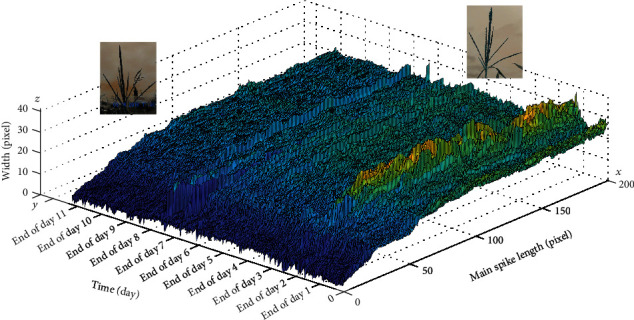
Flowering pattern of a representative tassel.

After removing the outliers, a 3D surf plot to monitor the width over time for one of the tassels in the camera is plotted, as shown in Figure 18. The *y*-axis represents images at different time points, the *x*-axis is the pixel location of the main spike, and the *z*-axis represents the width of the main spike. As can be seen in the figure, greater width indicates flowering. Notice that the location of the flowering began from the middle of the tassel and continued toward the bottom and the top of the main spike. This flowering happened over 2 days.

**Figure 19:**
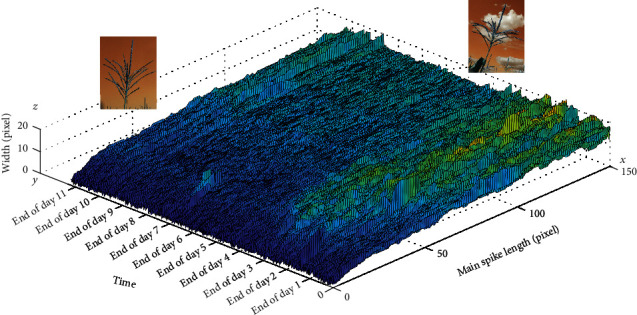
Flowering pattern of another representative tassel.

The flowering pattern of the other tassel captured by the same camera is shown in Figure 19 as a 3D surface plot. For this figure, the flowering took 3 days. In addition, according to these two plots, the flowering starts from the middle of the main spike and then evolves upward and downward, respectively [48].

## 4. Conclusion

The goal of this case study was to develop an automated sequential pipeline for investigating maize tassel flowering. We show that a workflow comprising several deep learning and image processing methods provides a robust end-to-end pipeline for this purpose. Tassel detection, classification, and segmentation modules were successfully trained and deployed. Such a sequential approach allows systematic course correction and minimizing the human annotation effort, which is especially promising for problems where creating the dataset is a major challenge. An outcome of this work is a workflow to extract the spatiotemporal map of anthesis progression. We are currently extracting data for a diverse set of genotypes, which will subsequently be used for a variety of downstream science analysis including GWAS. Finally, we emphasize that the use of deep learning architectures in plant phenotyping is a rapidly evolving discipline. Since the submission of this paper, we have made significant progress in using more end-to-end approaches (using the data from these models). Newer architectures [49, 50], automated hyperparameter exploration [51], semisupervised/self-supervised approaches [52, 53], and domain adaptation approaches [54] can be leveraged to provide improved results.

## Data Availability

Data will be made available on request.

## Supplementary Materials

Supplementary Materials Figure 1: RetinaNet object detection architecture. Figure 2: precision-recall curve obtained after testing the model. Figure 3: accuracy and loss value changes with every epoch of the classification model. Figure 4: accuracy and loss value changes with every epoch of the segmentation model. Figure 5: the flowchart of calculating the branch points of a tassel in a binary image.
