# Supplementary material for: Detection of the Progression of Anthesis in Field-Grown Maize Tassels: A Case Study
Source: Plant Phenomics. 2021 Mar 3;2021:4238701. doi: 10.34133/2021/4238701 (PMC7953991; doi:10.34133/2021/4238701)
Supplement: Supplementary Materials — Figure 1: RetinaNet object detection architecture. Figure 2: precision-recall curve obtained after testing the model. Figure 3: accuracy and loss value changes with every epoch of the classification model. Figure 4: accuracy and loss value changes with every epoch of the segmentation model. Figure 5: the flowchart of calculating the branch points of a tassel in a binary image. [file 4238701.f1.zip › supplementary.docx]

**Detection**

RetinaNet is a one-stage detector. Figure 1 shows the architecture of the model. Both classification and regression networks are merged into layers of features called pyramid of features. The outputs of these layers are passed to the regression and classification networks. All these predictions were combined by implementing non-maximum suppression. ResNet50 was used as a backend for training the network and ImageNet weights were loaded for initializing the weights as transfer learning. Focal loss was used to train the negative samples with less weights. Adam with learning rate of 0.0001 was used for optimizing the weights. The model was trained for 50 epochs and the batch size was 8. The model was trained by a GPU manufactured by NVIDIA Tesla V100-PCIE with 32 GB memory installed on the NOVA cluster at Iowa State University.

| 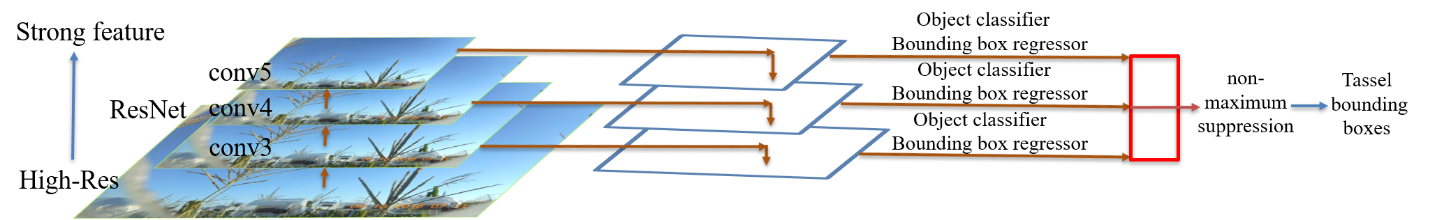 |
| --- |
| Figure 1. RetinaNet object detection architecture |

The model was test using the testing the dataset and mAP. Figure 2 shows the precision-recall curve for testing the model.

|  |
| --- |
| Figure 2. Precision-recall curve obtained after testing the model. |

**Classification**

RGB images with size of 387 × 516 × 3 used as an input image. 28 filters with size of 3 by 3 were used for each of the convolutional layers. After each convolutional layer, there was a max-pooling layer to reduce the computation load of the network and monitor the important features. The activating function was Rectified Linear Unit (ReLU) due to improvement in training speed. The 2D arrays were flattened to enable SoftMax for classifying the images as tassel or non-tassel. ADAM was A dropout was also added to the model to prevent from overfitting. Adam used as an optimizer and loss function was binary cross-entropy. The model was trained a GPU manufactured by NVIDIA Tesla V100-PCIE with 32 GB memory.

| 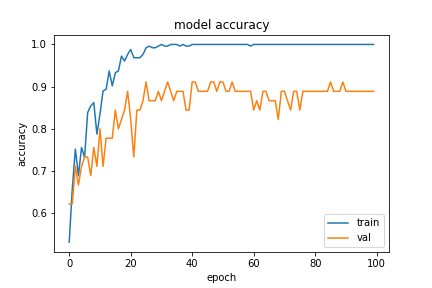 | 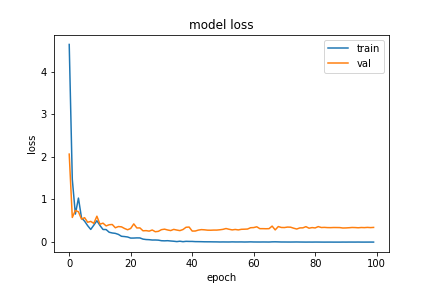 |
| --- | --- |
| Figure 3. Accuracy and loss value changes with every epoch of the classification model | |

**Segmentation**

A CNN-based segmentation was deployed to obtain binary image of each RGB image of tassels. This Autoencoder network has 4 encoding and 4 decoding layers. Size of input images were 792 × 612. Encoding layers have 2 convolutional layers, each followed by polling of (2 ×2). Size of convolutional layers were 3 × 3. The encoding section compress the input features using three convolutional layers and two pooling layers by taking the advantage of Relu function [62] as an activation function. The pooling layers were used to reduce the computational load. In decoding section, the same architecture was used such that the features can produce the corresponding binary image. To do so, ‘Adam’ and binary cross entropy were used as optimization and loss functions. The model was trained on the Nova cluster On GPU node (NVIDIA) at Iowa state university. The model was trained for 100 epochs. shows the accuracy and loss values over training for different epochs.

| 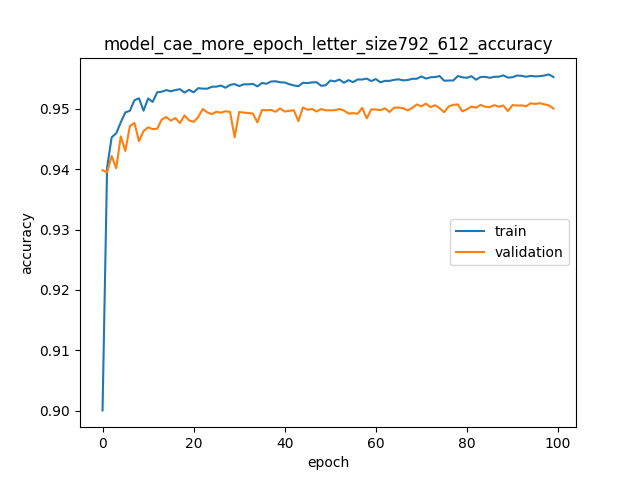 | 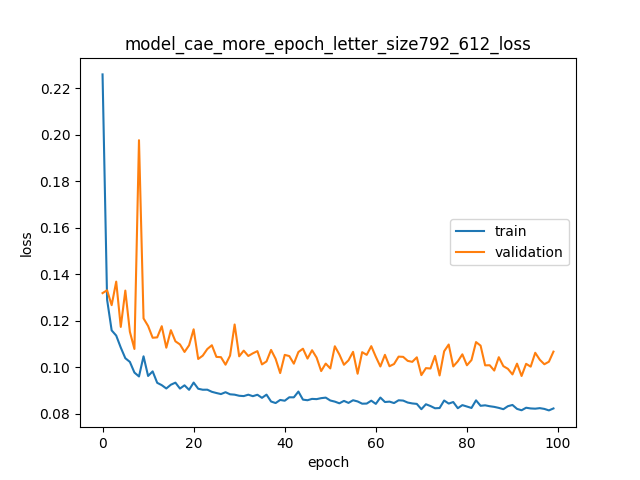 |
| --- | --- |
| Figure 4. Accuracy and loss value changes with every epoch of the segmentation model | |

**Tassel Analysis**

Once the binary images are obtained, a heuristic image processing method was used to analyze the images. First, the main spike of the tassel should be extracted from the tassel. Main spike is the longest branch of the tassel starting from the topmost branch point up to the tassel tip. Sometimes the main spike is covered with other tassel branches. So, we need to extract the visible part of the main spike such that we can track the flowering. To do so, the branch points and tip of each branches of the tassel were obtained. Figure 5 shows the flow chart of obtaining branch points and their tips.

|  | |
| --- | --- |
| Figure 5. The flowchart of calculating the branchpoints of tassel in a binary image |  |
