# Supplementary figures and images for: Detection of the Progression of Anthesis in Field-Grown Maize Tassels: A Case Study
Source: Plant Phenomics. 2021 Mar 3;2021:4238701. doi: 10.34133/2021/4238701 (PMC7953991; doi:10.34133/2021/4238701)

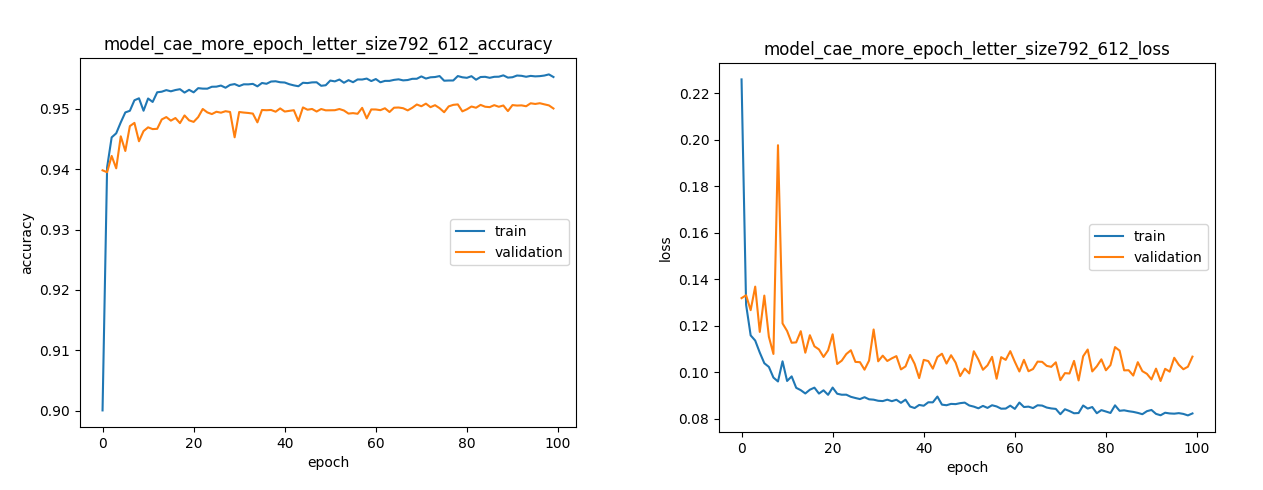

Supplement: Supplementary Materials — Figure 1: RetinaNet object detection architecture. Figure 2: precision-recall curve obtained after testing the model. Figure 3: accuracy and loss value changes with every epoch of the classification model. Figure 4: accuracy and loss value changes with every epoch of the segmentation model. Figure 5: the flowchart of calculating the branch points of a tassel in a binary image. [file 4238701.f1.zip › supp_figure_4.png]

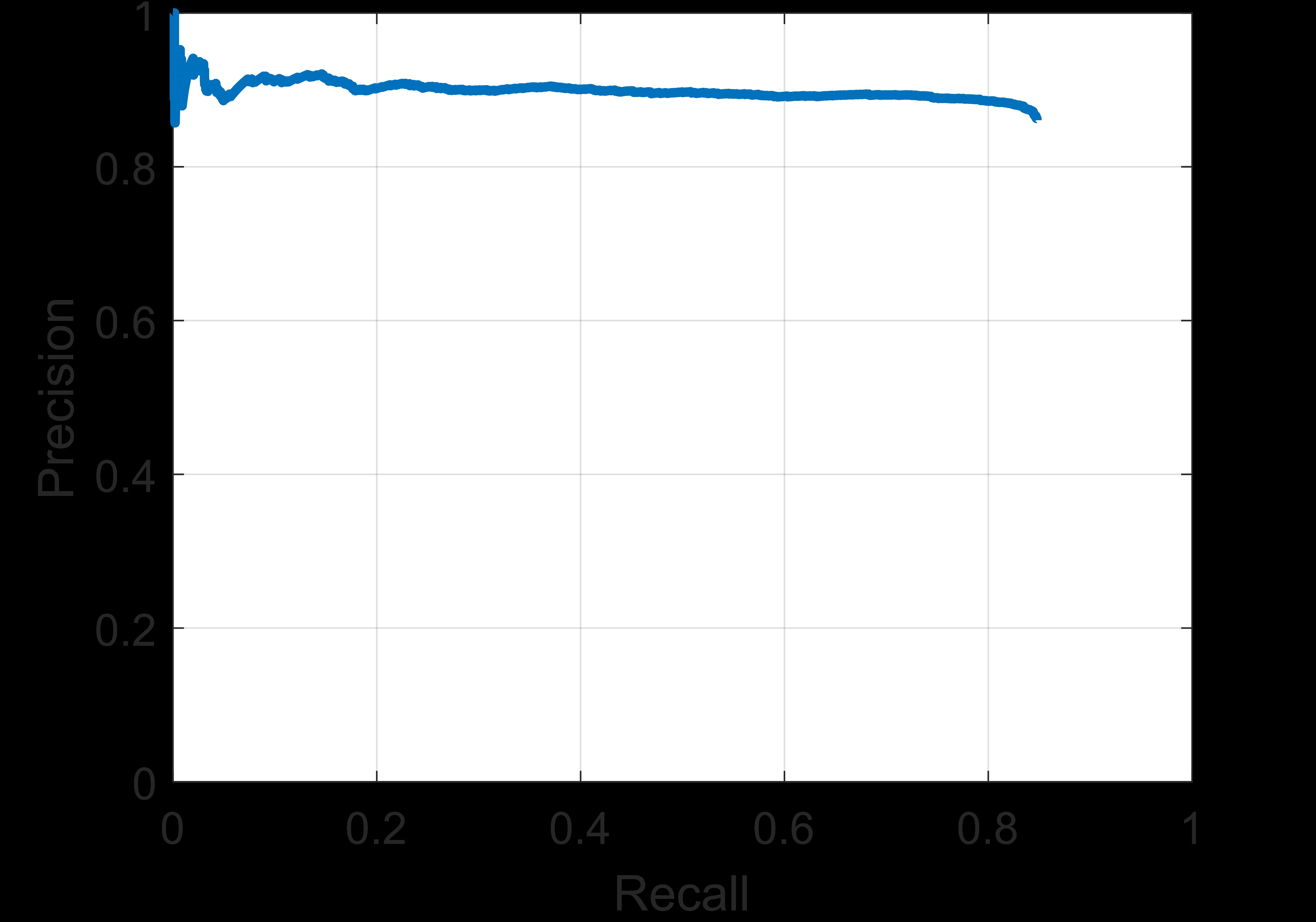

Supplement: Supplementary Materials — Figure 1: RetinaNet object detection architecture. Figure 2: precision-recall curve obtained after testing the model. Figure 3: accuracy and loss value changes with every epoch of the classification model. Figure 4: accuracy and loss value changes with every epoch of the segmentation model. Figure 5: the flowchart of calculating the branch points of a tassel in a binary image. [file 4238701.f1.zip › supplementaray_figure_2.jpg]

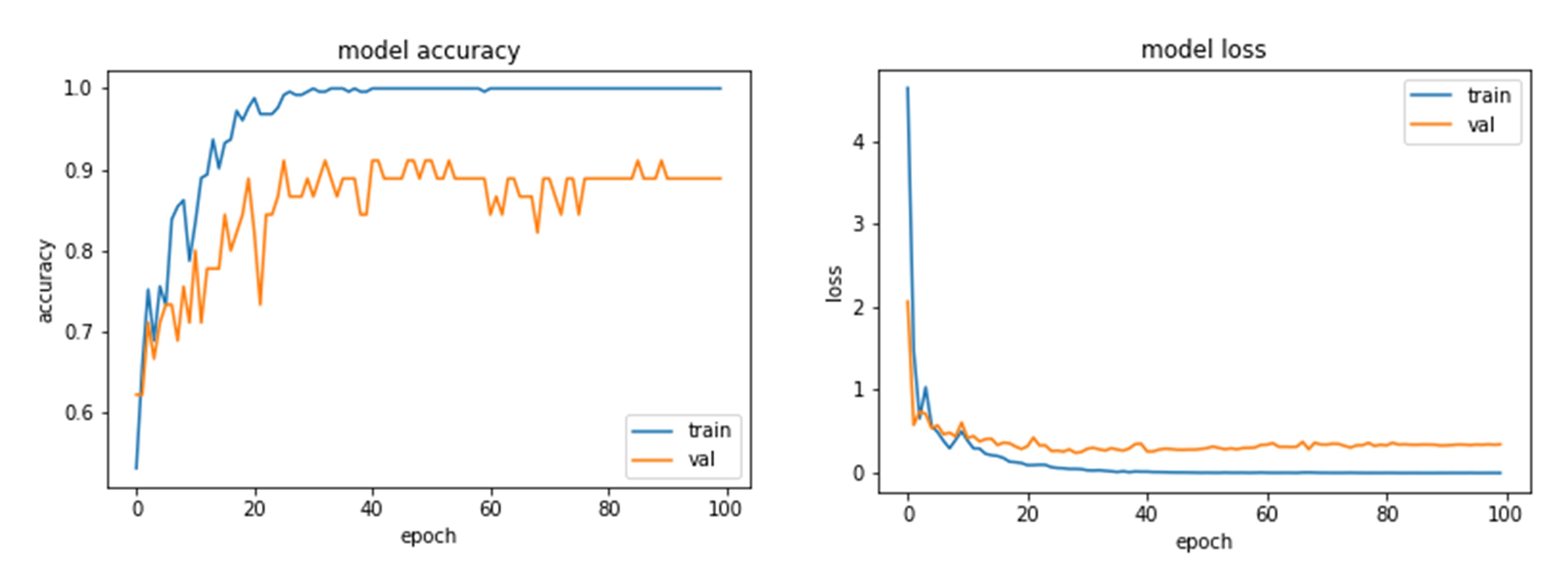

Supplement: Supplementary Materials — Figure 1: RetinaNet object detection architecture. Figure 2: precision-recall curve obtained after testing the model. Figure 3: accuracy and loss value changes with every epoch of the classification model. Figure 4: accuracy and loss value changes with every epoch of the segmentation model. Figure 5: the flowchart of calculating the branch points of a tassel in a binary image. [file 4238701.f1.zip › supplementaray_figure_3.jpg]
