# Supplementary material for: Detection of the Progression of Anthesis in Field-Grown Maize Tassels: A Case Study
Source: Plant Phenomics. 2021 Mar 3;2021:4238701. doi: 10.34133/2021/4238701 (PMC7953991; doi:10.34133/2021/4238701)

Theme

Color can add clarity and elegance. Pick a theme from the Design tab.

Background

Give your drawing a professional background. Select one on the Design tab.

Hover

Hover on a shape, then use AutoConnect arrows to connect new shapes.

Finished with these tips?
Select the Tip Pane and press Delete

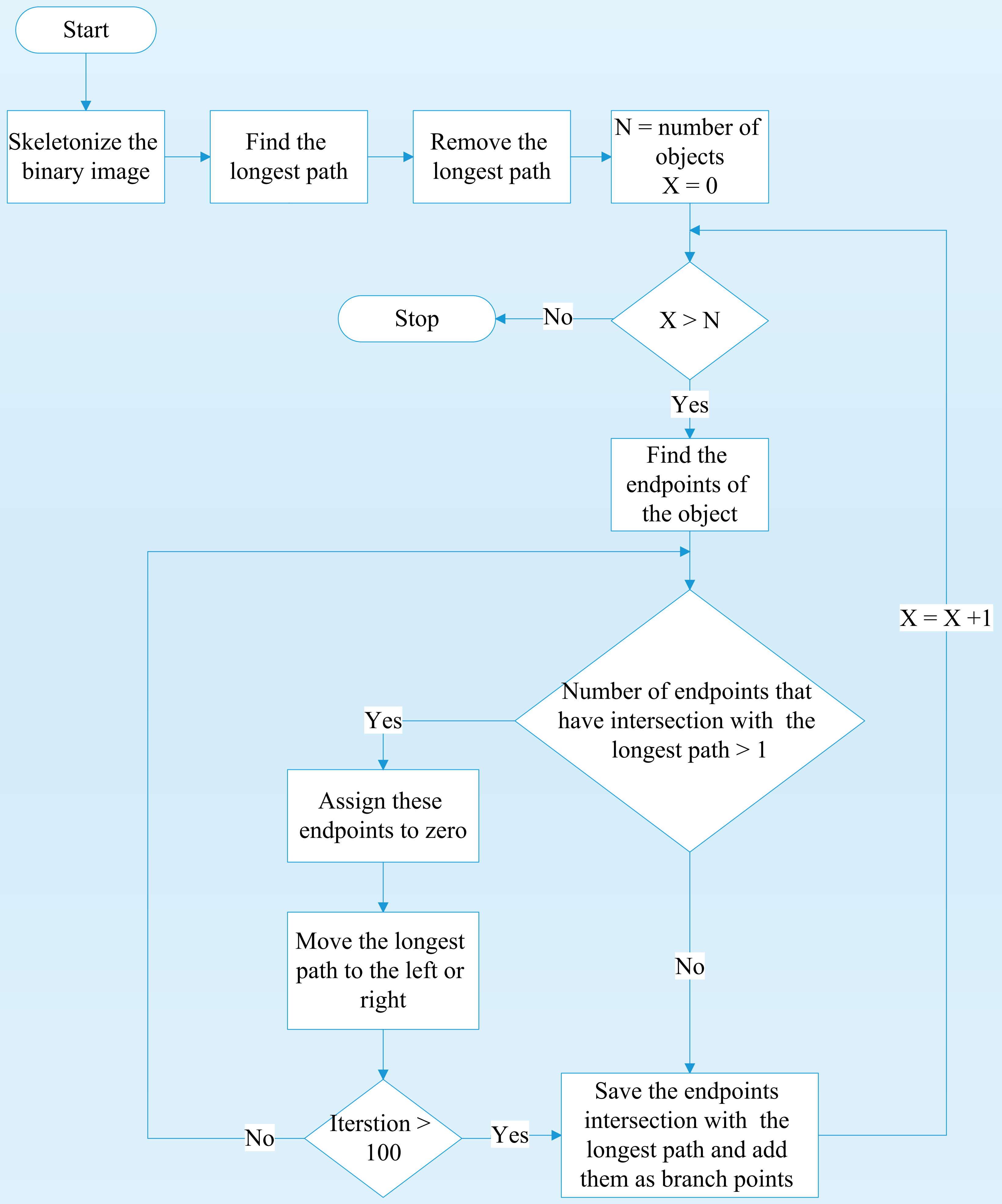

Supplement: Supplementary Materials — Figure 1: RetinaNet object detection architecture. Figure 2: precision-recall curve obtained after testing the model. Figure 3: accuracy and loss value changes with every epoch of the classification model. Figure 4: accuracy and loss value changes with every epoch of the segmentation model. Figure 5: the flowchart of calculating the branch points of a tassel in a binary image. [file supplementary_figure_5.pdf › supplementary_figure_5.pdf]
